# Children’s foot parameters and basic anthropometry — do arch height and midfoot width change?

**DOI:** 10.1007/s00431-022-04715-1

**Published:** 2022-12-07

**Authors:** Carles Escalona-Marfil, Anna Prats-Puig, Xavier Ortas-Deunosajut, Raquel Font-Lladó, Xavier Ruiz-Tarrazo, Angela Margaret Evans

**Affiliations:** 1 Sport, Exercise, and Human Movement (SEaHM) Research Group, Faculty of Health Sciences at Manresa, University of Vic - Central University of Catalonia (UVic-UCC), Av. Universitària, 4-6, 08242 Manresa, Spain; 2 University School of Health and Sport (EUSES), University of Girona, C/ Francesc Macià, 65, 17190 Salt, Girona, Spain; 3 Research Group of Clinical Anatomy, Embryology and Neuroscience (NEOMA), Department of Medical Sciences, University of Girona, 17003 Girona, Spain; 4 University of Girona, Cultura i Educació Research Group, Girona, Spain; 5 Discipline of Podiatry, School of Allied Health, Human Services and Sport, College of Science, Health and Engineering, La Trobe University, Plenty Road, Bundoora, Melbourne, 3086 Australia; 6 Faculty of Medicine and Health Sciences, School of Podiatry, Universitat de Barcelona, Barcelona, Spain

**Keywords:** Children, Weight status, Body composition, Foot posture, Foot morphology, Foot posture index, Arch height index, Midfoot width

## Abstract

The aims of this study were as follows: (1) to assess how foot posture and morphology assessments change according to body mass index (BMI) status; (2) to determine which body composition parameter (BMI or waist circumference) correlates better with the foot posture index (FPI), arch height index (AHI), and midfoot width (MFW) in children. Foot morphometry (FPI, AHI, and MFW) and body composition (BMI and waist circumference (WC)) were assessed in a cross-sectional study of 575 children (mean age = 7.42 ± 1.67 years; 53.27% female). When comparing BMI groups, an increase of 8.3% in AHI and 13.6% in MFW (both *p* < 0.0001) was seen. In linear regression analyses, BMI and WC were positively associated with MFW explaining together 64.8% of its variance. Noteworthy, MFW is the most related to body composition parameters.

*Conclusion*: Foot morphology assessed by FPI, AHI, and MFW differs among BMI categories in children. Noteworthy, WC correlates better with foot measures than does the more commonly used BMI, and more importantly the MFW is the foot measure best explained by children’s body weight. Since foot morphometry is different among different BMI groups, children would benefit from shoes with different patterns (thinner and wider), as well as a good system to adjust midfoot height.

**Table:** 

**What is Known:** • *Children who are overweight and obese have flatter feet, when assessed using footprints.* • *Up to 72% of people have incorrectly fitted shoes.*	
**What is New:** *• Children with underweight have thinner and flatter feet than children with normal weight, while children with overweight and obesity have wider and higher arched feet.* *• Body weight is related to foot shape, which has relevance for footwear manufacturers. *

**What is Known:**

• *Children who are overweight and obese have flatter feet, when assessed using footprints.*

• *Up to 72% of people have incorrectly fitted shoes.*

**What is New:**

*• Children with underweight have thinner and flatter feet than children with normal weight, while children with overweight and obesity have wider and higher arched feet.*

*• Body weight is related to foot shape, which has relevance for footwear manufacturers.
*

## Introduction

Obesity in children has been related with changes in the musculoskeletal system, lower limb functionality [1], bone microarchitecture [2], increased risk of suffering lower limb pain or fractures [3–5], and increased plantar pressure [6, 7]. In the same way, underweight in children is also related to an increased risk of bone fractures [8] and according to Mauch et al. a specific foot morphology (slender and long feet) [9]. The feet transmit body load to the ground through a complex bone and soft tissue structure. Despite several previous studies hypothesizing that children with an increased fat mass have a flatter foot, how body weight and composition affect foot posture and morphology needs further study.

Foot posture can be assessed in children with several measures and indices. The foot posture index (FPI) is a global measure of 6 foot parameters, which is assessed by palpation and observation, allowing to categorize the feet into 5 groups, from highly supinated to highly pronated (or flat foot); the Staheli arch index (AI) and Chippaux-Smirak index (CSI) are assessed using footprints. The three outcomes have shown to be reliable and hence recommended for research purposes [10]. Recently, two alternative, single, direct anthropometric measures of foot morphology have demonstrated to be reliable in children: the arch height index (AHI) — also called arch height ratio (AHR) — and the midfoot width (MFW) [11–13].

Conflicting data exist regarding to what extent overweight and obesity is causing or related to flatfoot. While some studies observed associations between foot measures such as FPI, AI, CSI, and weight or BMI [14–22], others did not [23–30]. Additionally, there is no previous literature regarding AHI and MFW and weight status in children.

The aims of this study were as follows: (1) to assess how foot posture and morphology assessments change according to BMI status; (2) to determine which anthropometric parameter (BMI or waist circumference) correlates better with the FPI, AHI, and MFW in children.

## Methods

A cross-sectional observational study was designed. Five hundred seventy-five healthy, asymptomatic children were recruited through the “Precocious Detection Program” (PDP) and the “Physical Education, Health and Children (PEHC)” [31] research programs in Manresa and Girona (Catalunya), respectively. Inclusion criteria were as follows: (1) age between 5 and 10 years old; (2) apparently healthy children. Exclusion criteria were as follows: (1) lower extremities congenital deformity or fractures; (2) neurological conditions that could alter the outcomes.

The research was approved by the Comitè Ètic d’Investigació (CEI) de la Fundació Unió Catalana d’Hospitals (code CEI 17/62) and the Institutional Review Board of Dr. Josep Trueta Hospital (code Competencia motricitat). Signed consent was obtained from the parents of all children.

Body composition measurements were performed by one expert observer, who was unaware of the foot assessments of the participants. Two measurements were made, and their average was calculated. Weight was measured with a calibrated scale (Portable TANITA; 240MA, Amsterdam, The Netherlands) in the morning before eating any food, and wearing light clothes, and height was measured using a wall mounted stadiometer (SECA SE206, Hamburg, Germany). Body mass index (BMI) for each participant was calculated using the formula: weight (kilograms) divided by the square of height in meters. BMI-SDS (standard deviation score) was standardized according to age- and sex-adjusted values from regional normative data [32]. Weight status groups were created as follows [33]: underweight, children with a BMI-SDS ≤  −1; normal weight, children with a BMI-SDS between −1 and 1; and children with overweight and obesity, with a BMISDS ≥ 1. Waist circumference was measured in the standing position at the umbilical level (SECA 203, Hamburg, Germany). Waist-SDS (standard deviation score) was standardized according to age- and sex-adjusted values from regional normative data [34].

Foot measures were performed twice for FPI, dorsal arch height (DAH), arch height index (AHI), and midfoot width (MFW) and the average was calculated. These were measured by two experienced observers, who were blinded to body measurements. Two measurements were performed, and the average was calculated. These were measured on the left foot by the same experienced observer [35, 36]. The FPI is a scaled measure of global foot pronation with values ranging from −12 (highly supinated) to + 12 (highly pronated) [37, 38]. The DAH is a single measure of the medial longitudinal arch (MLA); using a digital caliper, the distance between the floor and the dorsum of the foot at 50% of total foot length is measured. The DAH is normalized to the total foot length, creating a ratio (DAH/foot length), obtaining the AHI. The MFW is assessed with a modified digital caliper at the 50% of foot length. All the measures have demonstrated to be reliable in a pediatric population [12, 39] and performed with children standing.

For data analysis, SPSS version 22.0 (SPSS Inc., Chicago, IL) was used. All data were tested for normality using a Kolgomorov-Smirnov test. FPI did not follow the normal distribution, and non-parametric test was applied when analyzing FPI. Differences between groups were analyzed by one-way ANOVA or Kruskal–Wallis test. The relations between variables were analyzed by Pearson or Spearman correlation followed by linear regression analysis using the stepwise method. Significance level was set at *p* = 0.0083 after multiple testing correction (0.05/6 comparisons).

## Results

A sample of 575 children (mean age = 7.42 ± 1.67 years; 53.27% female) was assessed. Table 1 shows the results for clinical and foot measurements, according to weight groups. We show that the three groups differ in weight, height, BMI, and waist (all *p* < 0.0001), as was expected. Moreover, a higher proportion of girls were underweight (*p* = 0.015) and children who were overweight and obese were slightly older (*p* = 0.022). Regarding foot assessments, FPI tended to decrease when comparing groups (*p* = 0.022). However, a higher BMI-SDS was related to an 8.3% increase in AHI and 13.6% increase in MFW (both *p* < 0.0001) when comparing groups.

**Table 1:** Clinical and foot assessments in children according to weight status groups (*n* = 575)

	Underweight (BMI-SDS ≤ − 1; *n* = 90)	Normal weight (BMI-SDS > − 1 and < 1; *n* = 416)	Overweight or obese (BMI-SDS ≥ 1) (*n* = 69)	*p* value
Clinical assessments				
Age (year)	7.48 ± 1.72	7.14 ± 1.63	7.65 ± 1.68	0.022
Gender (%Female)	60.0	44.7	55.1	0.015
Weight (kg)	21.5 ± 4.7	25.3 ± 6.2	37.3 ± 10.0	< 0.0001
Weight-SDS	− 1.06 ± 0.57	− 0.20 ± 0.62	1.63 ± 0.87	< 0.0001
Height (m)	1.24 ± 0.11	1.23 ± 0.11	1.29 ± 0.12	< 0.0001
Height-SDS	0.00 ± 1.37	− 0.05 ± 1.11	0.65 ± 1.09	< 0.0001
BMI (kg/m^2^)	13.6 ± 1.1	16.5 ± 1.4	21.7 ± 2.2	< 0.0001
BMI-SDS	− 1.38 ± 0.43	− 0.21 ± 0.50	1.67 ± 0.61	< 0.0001
Waist circumference (cm)*	52.9 ± 2.9	58.9 ± 4.4	73.7 ± 6.0	< 0.0001
Waist-SDS*	− 1.07 ± 0.56	− 0.02 ± 0.89	2.50 ± 1.29	< 0.0001
Foot assessments				
FPI — left^¥^	4 (3–7)	4 (3–6)	3 (2–5)	0.022
AHI — left	0.243 ± 0.021	0.255 ± 0.016	0.261 ± 0.017	< 0.0001
MFW — left (mm)	61.4 ± 6.1	64.0 ± 6.2	69.8 ± 7.1	< 0.0001

*Waist circumference was assessed in a subgroup of 320 children

Normally distributed data are shown as mean ± SD. ^¥^Non-normally distributed data are shown as mean (interquartile range). *BMI* body mass index, *SDS* standard deviation score, *FPI* foot posture index, *AHI* arch height index, *MFW* midfoot width. *p* values are from one-way ANOVA or Kruskal–Wallis test

**Fig. 1:**
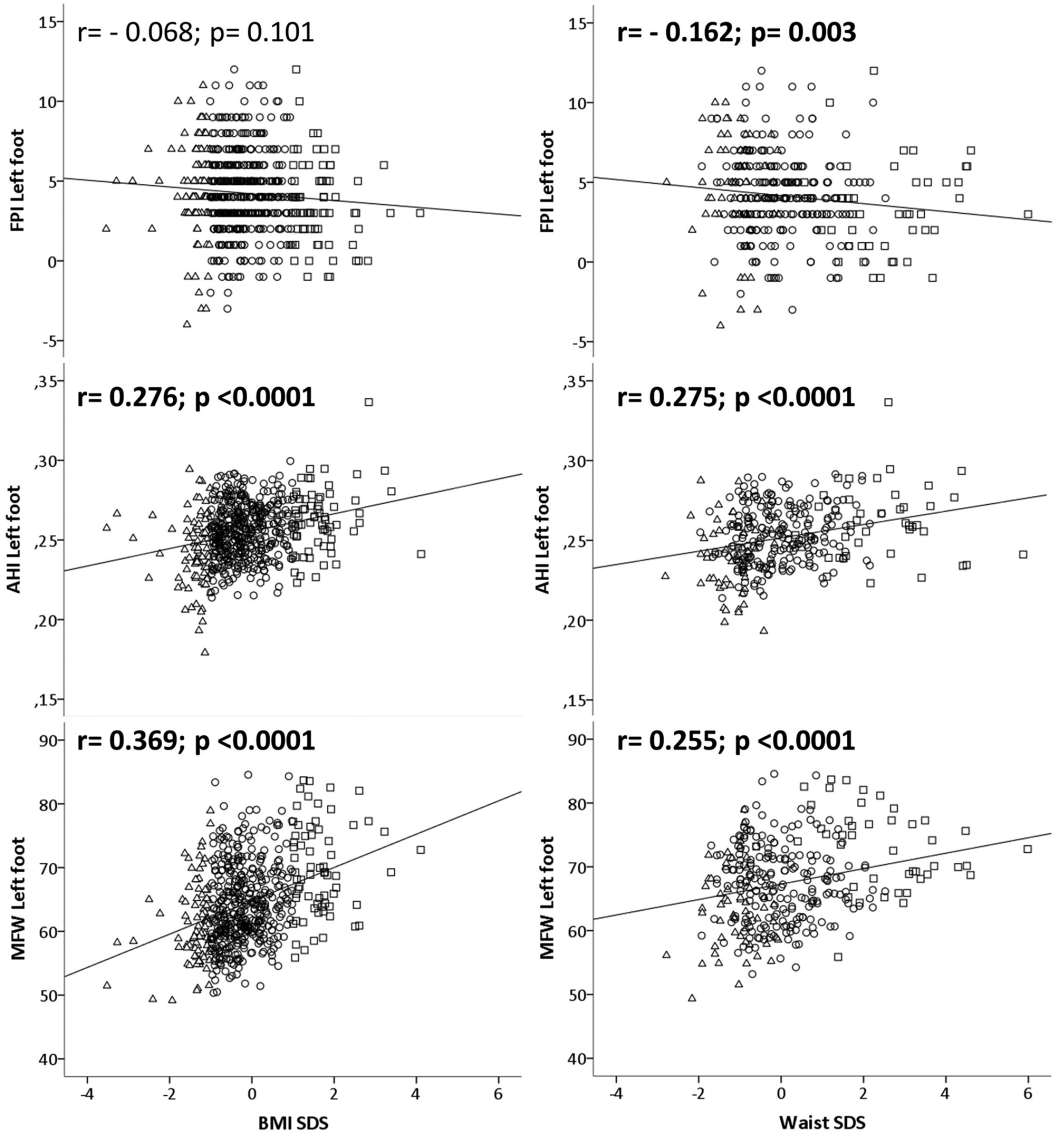
Scatter plots for FPI, AHI, and MFW with **A** children’s BMI-SDS (*n* = 575) and **B** children’s waist-SDS (*n* = 320). Squares depict children with obesity (BMI-SDS ≥ 1), dots depict lean children (BMI-SDS >  −1 and < 1), and triangles depict children with underweight (BMI-SDS ≤  −1). *p* values are from Pearson or Spearman correlation

Figure 1 depicts correlations between foot measurements (FPI, AHI, and MFW) and BMI-SDS and WC-SDS. WC-SDS correlates with all foot measurements (negatively with FPI (*r* =  −0.162; *p* = 0.003); positively with AHI (*r* = 0.275; *p* < 0.0001) and MFW (*r* = 0.255; *p* < 0.0001)), while BMI-SDS correlates positively with AHI (*r* = 0.276; *p* < 0.0001) and MFW (*r* = 0.369; *p* < 0.0001), but not with FPI (*r* =  −0.068; *p* = 0.101). All significant associations were still relevant after correcting for multiple testing.

In linear regression analyses after adjusting for gender and age, BMI was independently associated with FPI, AHI, and MFW explaining, 1.6%, 14%, and 65.6% of its variability respectively (Table 2, part A). After correcting for multiple testing, only AHI and MFW were still significant. In the same way, WC was independently associated with FPI, AHI, and MFW explaining 2.5%, 12.1%, and 62.3% of its variability respectively (Table 2, part B); all associations remained significant after multiple testing correction. Both BMI and WC were independently and positively associated with MFW explaining together 64.8% of its variance, after adjusting for age and sex (Table 2, part C). When assessing BMI and WC together, the strongest association with FPI was seen for WC (*β* =  −0.247, *p* = 0.035, and *R*^2^ = 0.023) and only BMI was independently associated with AHI (*β* = 0.364, *p* = 0.001, and *R*^2^ = 0.150). Noteworthy, FPI is the most poorly explained by both BMI and WC, while the MFW is the most related to both body composition parameters. However, only after correcting for multiple testing, BMI remained significant.

**Table 2:** Linear regression analyses for FPI, AHI, and MFW as dependent variables (*n* = 575). (A) BMI as independent variable; (B) WC as an independent variable; (C) BMI and WC as independent variables

	FPI		AHI		MFW	
	*β*	*P*	*β*	*p*	*β*	*p*
A)						
Gender	− 0.092	0.025	− 0.115	0.003	− 0.170	< 0.0001
Age	− 0.037	0.390	− 0.306	< 0.0001	0.561	< 0.0001
BMI	− 0.093	0.030	0.309	< 0.0001	0.415	< 0.0001
Total *R*^2^	0.016		0.140		0.656	
B)						
Gender	− 0.026	0.624	− 0.119	0.024	− 0.161	< 0.0001
Age	− 0.017	0.761	− 0.229	< 0.0001	0.558	< 0.0001
WC	− 0.180	0.001	0.285	< 0.0001	0.458	< 0.0001
Total *R*^2^	0.025		0.121		0.623	
C)						
Gender	− 0.027	0.625	− 0.120	0.021	− 0.168	< 0.0001
Age	− 0.025	0.659	− 0.263	< 0.0001	0.529	< 0.0001
BMI	0.078	0.512	0.364	0.001	0.356	< 0.0001
WC	− 0.247	0.035	− 0.026	0.812	0.141	0.048
Total *R*^2^	0.023		0.150		0.648	

*BMI* body mass index, *WC* waist circumference, *FPI* foot posture index, *AHI* arch height index, *MFW* midfoot width

*p* values are from linear regression analysis using the stepwise method

## Discussion

A cross-sectional study was conducted in 575 healthy children aged 5 to 10 years. Body mass was rated with BMI and WC (both widely used in clinical practice) [40]. Foot posture and morphology were compared and contrasted using three measures: FPI, AHI, and MFW. The main findings were that foot morphology is different according to weight status of the child. Children with underweight have thinner and flatter feet than children with normal weight, while children with overweight and obesity have wider and higher arch feet.

The relationship between BMI and foot posture has long produced dissent [41]. Since no consensus has been achieved regarding the importance of body weight in foot posture, this situation challenges clinicians when assessing children with overweight and obesity and flat feet [42]. Several studies demonstrate that children who are overweight and obese have flatter feet, when assessed using footprints [15–22]. However, when comparing global foot posture with the FPI instead of a footprint, other authors have found that this relationship does not exist [14, 23–30]. Our results show that FPI is poorly related to BMI and WC. However, when observing single foot measures such as AHI (a normalized index independent of foot size), AHI is lower in underweighted children and higher in children with overweight and obesity, indicating that heavier children have higher arches. Moreover, heavier children also present a wider foot, represented by an increased MFW. Taken together, we hypothesize that children with overweight and obesity may have thicker plantar fat pad and greater foot adiposity, but more studies are needed since a consensus has not been reached [19, 43].

Although our results showed that FPI is poorly explained by both BMI and WC, and hence the foot posture may be quite independent from body weight, children with higher BMI or wider waist tend to have lower FPI values (less pronated or flat feet), while children with underweight had more pronated feet. When assessing single foot measures, both AHI and MFW correlate positively with BMI and WC. From a clinical perspective, it means that children with higher weight have higher arches and wider feet. To what extent the arch height is due to the fatness remains unclear, as Mickle et al. found that the thickness of the midfoot plantar fat pad was not different between normal weight and overweight children [19], while Riddiford-Harland et al. reported that obese children had fatter and flatter feet [43]. To be noted, while the FPI is an index and AHI is a ratio, the MFW is a real length measure (mm).

WC is an anthropometric measure related to metabolic and clinical disorders, and identifies the risk of cardiovascular diseases, more readily than BMI. However, previous studies have focused more on BMI than WC, when analyzing the relationship between obesity and foot outcomes. To the authors’ best knowledge, this is the first study to examine and identify the relationship between WC and foot posture in healthy children, and to make comparison with BMI [44].

When assessing the pediatric foot, few measures have demonstrated adequate reliability [10]. The FPI has been widely used in scientific literature; however, it may not be commonly used in clinical practice, especially by non-podiatrists. AHI and MFW are single, reliable anthropometric measures that are easier for clinicians to observe and to use. Interestingly, we found that both AHI and MFW correlated to children’s body weight, as measured by both BMI and WC. Hence, when exploring children who are overweight and obese, one should expect increased dorsal arch height and, especially, wider feet to be considered normal, while underweight children (a less studied issue, but also with significant differences) [9] usually have lower dorsal arch height and thinner feet.

It has been previously stated that ill-fitting footwear is commonly related to foot pain and foot disorders, and that up to 72% of people have incorrectly fitted shoes [45]. This also applies to children, as most have poorly fitted shoes, as detected in southern Spain, where 72.5% of children were found to wear shoes too short, and 66.7% wore shoes too narrow [46] and in Poland, where about 40% of the girls analyzed wore too short shoes, while nearly 50% wore them too wide [47]. In addition to other health implications, body weight is related to foot shape, which has relevance for footwear manufacturers. Manufacturers should be aware that children’s feet do differ not only in length, but also in dorsal arch height (“instep”) and midfoot width. As a result, different patterns should be offered, as well as the possibility of easily adjustable styles.

This study had some limitations. The sample body weight distribution was proportioned as the following: normal weight (72.35%), underweight (15.65%), overweight and obesity (12%). While this reflects the studied population, future studies could recruit more homogeneous group sizes. Similarly, the children’s age ranged from 5 to 10 years; hence, the results are limited to these ages. Future studies with larger sample size should elucidate possible foot differences when comparing children with overweight and with obesity, and in older age groups.

## Conclusions

In conclusion, the results of this investigation reveal that foot measures (FPI, AHI, and MFW) differ among children with normal weight and children with overweight and obesity, and also in children with underweight. In addition, WC correlates better with foot measures than does BMI, and more importantly the MFW is the foot measure best explained by children’s body weight. Since foot morphometry is different among different BMI groups, children would benefit from shoes with different patterns (thinner and wider), as well as a good system to adjust midfoot height.

## Abbreviations

BMI: Body mass index
WC: Waist circumference
FPI: Foot posture index
AHI: The arch height index
MFW: Midfoot width
SDS: Standard deviation score
DAH: Dorsal arch height
